# The Impact of *UGT1A1* Genetic Variability on Enzyme Expression in Liver Pathology

**DOI:** 10.3390/genes17050589

**Published:** 2026-05-21

**Authors:** Sylwia Szeląg-Pieniek, Joanna Kucak, Damian Malinowski, Stefan Oswald, Marek Droździk, Mateusz Kurzawski

**Affiliations:** 1 Department of Experimental and Clinical Pharmacology, Pomeranian Medical University, Al. Powstańców Wlkp. 72, 70-111 Szczecin, Poland; sylwia.szelag.pieniek@pum.edu.pl (S.S.-P.); marek.drozdzik@pum.edu.pl (M.D.); 2 Independent Pharmacodynamics Laboratory, Pomeranian Medical University, pl. Polskiego Czerwonego Krzyża 1, 71-244 Szczecin, Poland; 3 Department of Pharmacokinetics and Monitored Therapy, Pomeranian Medical University, Al. Powstańców Wlkp. 72, 70-111 Szczecin, Poland; damian.malinowski@pum.edu.pl; 4 Institute of Pharmacology and Toxicology, Rostock University Medical Center, 18057 Rostock, Germany; stefan.oswald@med.uni-rostock.de

**Keywords:** Gilbert’s syndrome, UDP-glucuronosyltransferase, glucuronidation, drug metabolism, liver pathology

## Abstract

Background: Glucuronidation is a major phase II biotransformation reaction responsible for the inactivation and elimination of endogenous compounds and pharmaceuticals. This process is catalyzed by enzymes belonging to the UDP-glucuronosyltransferase (UGT) family, among which UGT1A1 is the most extensively studied. UGT1A1 plays a critical role in the metabolism of numerous drugs and is essential for bilirubin glucuronidation. Due to this specific function, it has significant clinical relevance, as impaired enzyme activity resulting from genetic polymorphisms or mutations can lead to the accumulation of unconjugated bilirubin in the bloodstream. This study aimed to evaluate the association between the UGT1A1*28 allele and the gene expression and protein levels in human liver tissue, in relation to different liver diseases. Methods: Liver tissues were obtained from patients with various liver pathologies and the control group consisted of tissues showing no pathological symptoms. A total of 143 patients were included in the study. Pyrosequencing was used to analyze PCR products for the UGT1A1*28 polymorphism (variable number of TA repeats in the TATA sequence of the promoter). Results: In both the control and study groups, *UGT1A1* gene expression at the mRNA level and protein concentration in liver tissue decreased with the increasing number of UGT1A1*28 alleles. The association between *UGT1A1* genotype and mRNA/protein content was most pronounced in HCV and AIH, and slightly weaker in the ALK and PBC groups. The significant association between UGT1A1*28 allele and bilirubin concentration was observed in control group but not in liver pathology. Conclusions: The UGT1A1*28 polymorphism is associated with reduced hepatic mRNA and protein abundance in patients with liver disease. However, the significance of this observation for the metabolism of the enzyme substrates should be further investigated.

## 1. Introduction

Maintaining homeostasis in the human body depends on the effective metabolism and elimination of potentially harmful compounds, including endogenous metabolic by-products, exogenous toxins and medications. It is evident that biochemical processes carried out by CYP450 enzymes and UDP- glucuronosyltransferase play a pivotal role in the process of detoxification. These reactions involve oxidation and conjugation with a polar endogenous compound, respectively [1,2].

Glucuronidation is the most prevalent among phase II of detoxification reactions in mammals. It is primarily catalyzed by enzymes belonging to the UGT (UDP-glucuronosyltransferase) family [3]. They are categorized within a superfamily of enzymes that are present in the cells of animals, plants, fungi, and bacteria. The reaction catalyzed by these enzymes plays a key role in the inactivation of metabolized substances. Furthermore, UGT enzymes facilitate the dissolution of substances in water, enabling their excretion from the body via bile or urine. The highest expression of genes encoding UGT enzymes has been observed in organs responsible for detoxification, such as the liver, kidneys, and intestines. However, these enzymes have also been detected in other tissues, including the stomach, skin, mucous membranes, breasts, and prostate [4,5,6,7]. Moreover, the expression of these genes can be induced by signaling pathways that respond to the demand for detoxification or modulation of endogenous signaling molecules. This highlights the important role of UGT enzymes in the body’s detoxification processes [8,9,10].

The UGT gene family members can be classified into four main groups: UGT1, UGT2, UGT3 and UGT8. These genes are responsible for encoding a total of 22 functional enzymes within the human body. The UGT1 and UGT2 families are the most significant groups of UDP-glucuronosyltransferases and have been extensively researched in the fields of pharmacology and toxicology [11]. While many UGT enzymes are involved in the metabolism of both exogenous and endogenous compounds, UGT1A1 is unique in that it is primarily responsible for bilirubin glucuronidation. This specific feature gives it particular clinical significance. Disruption to the function of the *UGT1A1* gene resulting from genetic variants can lead to the accumulation of unconjugated bilirubin in the blood. This can result in benign or fatal inherited types of bilirubin metabolism disorders, such as Gilbert’s syndrome and Crigler–Najjar syndrome [7,12].

The spectrum of *UGT1A1* variants varies markedly. The most clinically important allele the in the Caucasian population is a TA insertion in the TATA-box sequence of the *UGT1A1* gene (allele *28). This variant results in seven repetitions A(TA)7TAA in the gene promoter, whereas the wild-type allele (UGT1A1*1) contains six repetitions [13]. The homozygous genotype of the UGT1A1*28 variant is associated with Gilbert’s syndrome, a benign inherited disorder characterized by decreased UGT1A1 enzyme activity by approximately 60–70% compared to the wild-type UGT1A1*1/*1 genotype. Symptoms are more prevalent in males than in females and manifest as diminished bilirubin conjugation capacity, which leads to mild hyperbilirubinemia [14]. Gilbert’s syndrome has a non-specific clinical presentation, and factors such as stress, sleep deprivation, alcohol consumption, dehydration, surgery, and the use of certain medications have been observed to exacerbate symptoms, including yellowing of the sclera and skin (without pruritus), as well as abdominal pain, nausea, vomiting, and flu-like symptoms. However, the syndrome is often asymptomatic, meaning up to 33% of affected individuals remain undiagnosed. The prevalence of the UGT1A1*28/*28 homozygous genotype varies across populations and is estimated at 2–15%. A recent study in the Czech Republic detected the UGT1A1*28/*28 genotype in 13.8% (99/717) of the general population, while Gilbert’s syndrome, defined by hyperbilirubinemia, was observed in 6.1% of females and 11.6% of males [15,16,17]. Individuals diagnosed with Gilbert’s syndrome typically do not require treatment; however, they should try to avoid factors that may exacerbate hyperbilirubinemia. Although Gilbert’s syndrome is most often associated with the UGT1A1*28 polymorphism, it is not the only variant underlying this condition. It is important to note that other alleles, including those involving mutations outside the promoter, must also be considered; however, these are rare in Caucasians [18].

In addition to genetic variability, the metabolic capacity of the UGT1A1 enzyme may be modulated by regulatory factors, including the activation of transcription factors and the influence of epigenetic factors [19,20,21]. The enzyme’s response to chemicals, both endogenous and exogenous, including drugs, may also be an important factor in regulating its activity [22,23,24].

In clinical practice, the UGT1A1*28 polymorphism resulting in reduced gene transcription and enzyme activity may lead to an increased risk of adverse reactions to drugs metabolized by UGT1A1, such as severe diarrhea and neutropenia following irinotecan administration [25,26] or elevated bilirubin levels during belinostat therapy [27,28]. To date, most of the available studies on the relationship between *UGT1A1* genetic variability and its function have focused on analyzing enzymatic activity. This has been assessed indirectly, either based on the glucuronidation rate of selected substrates [29,30], or based on serum bilirubin concentration as an indicator of glucuronidation capacity [31]. Direct measurement of the transcript and enzyme protein content in human livers has been much less common, due to the limited availability of liver biopsies for research purposes. Only a few studies that included both mRNA and protein measurements have been performed, and these were limited to specific age and ethnic groups. Additionally, most of these studies did not consider the clinical context, such as the presence or severity of liver disease, nor additional factors, such as environmental factors [32,33]. Therefore, it remains unclear whether the relationship between *UGT1A1* polymorphisms and gene expression is preserved across different liver diseases.

To ensure both the safety and efficacy of pharmacotherapy, a comprehensive understanding of the impact of common genetic variants on drug metabolism mediated by UGT1A1 is essential. Given that impaired drug metabolism is a characteristic of liver disease, genetic polymorphism may be an important factor in modifying drug biotransformation. The current study aimed to investigate the relationship between the UGT1A1*28 allele and *UGT1A1* gene expression, as well as UGT1A1 protein concentration, in human liver tissue across different liver diseases.

## 2. Materials and Methods

### 2.1. UGT1A1*28 Allele Status Determination

Liver samples were collected from patients undergoing elective liver transplantation and diagnosed with hepatitis C virus (HCV, *n* = 28), primary biliary cholangitis (PBC, *n* = 11), primary sclerosing cholangitis (PSC, *n* = 5), alcoholic liver disease (ALD, *n* = 19), Wilson’s disease (WD, *n* = 6) or autoimmune hepatitis (AIH, *n* = 17). Control samples were taken from non-tumorous liver tissue of patients undergoing resection of metastatic tumors (*n* = 28). All samples were collected and stored as previously described [34,35,36]. The clinical characteristics of the study participants are given in Supplementary Table S1. The study was carried out in accordance with the Declaration of Helsinki and the study protocol was approved by the local Bioethics Committee at the Pomeranian Medical University (approval number KB-006/06/2025). Informed consent was obtained from all participants. Genomic DNA was extracted from liver samples by means of GeneMATRIX Tissue DNA Purification Kit (EURx, Gdańsk, Poland) according to the manufacturer’s protocol. The UGT1A1*28 allele status was determined by means of pyrosequencing, using PyroMark Q48 Autoprep instrument (Qiagen, Redwood City, CA, USA) and primers (Genomed, Warszawa, Poland) previously described by Sukasem et al. [37]. DNA template was prepared using PyroMark PCR Kit (Qiagen, Hilden, Germany), pyrosequencing was performed using PyroMark Q48 Advanced Reagents (Qiagen, Hilden, Germany) according to the manufacturer’s protocol.

### 2.2. Gene Expression and Protein Quantification of UGT1A1

The methods used to measure gene expression (qPCR) and the abundance of the UGT1A1 enzyme protein (LC-MS/MS) in liver pathologies and control samples have been published previously [34,35].

In brief, total RNA was isolated from 20 mg of tissue specimen using Direct-zol RNA MiniPrep kit (Zymo Research, Irvine, CA, USA). Sample storage times varied from 1 to 42 months. Reverse transcription was performed using SuperScript VILO Master Mix (Thermo Fisher Scientific, Waltham, MA, USA), with 500 ng of total RNA in a reaction volume of 20 µL, according to the supplier’s protocol. The relative expression of the *UGT1A1* gene was determined using real-time PCR with a QuantStudio 7 Pro Real-Time PCR System (Life Technologies, Waltham, MA, USA). Threshold values were set manually at 0.1 for all genes. CT values were used to calculate relative transcript concentrations (ΔCT method). The mean CT value of the five housekeeping genes: GAPDH, PPIA, HMBS, RPLP0, and RPS9, was calculated and used as a reference to quantify the relative expression of the investigated gene. Details of the TaqMan assays are provided in Supplementary Table S2.

Quantification of the UGT1A1 protein was performed using a validated LC–MS/MS method based on targeted proteomics and mass spectrometry. In brief, about 40 mg of pulverized tissue was added to 1 mL of lysis buffer (0.2% SDS, 5 mM EDTA) containing 5 µL/mL Protease Inhibitor Cocktail (ProteoExtract-Native Membrane Extraction Kit; Merck, Darmstadt, Germany). The mixture was then homogenized manually using a Dounce homogenizer and incubated for 30 min at 4 °C. After determining the protein concentration using the Pierce BCA Protein Assay Kit (Thermo Fisher Scientific), a volume corresponding to 100 µg of protein was subjected to the established filter-aided sample preparation method. The obtained data were normalized to the respective mass of tissue lysate used in the tryptic digest. LC–MS/MS analyses were conducted on API4000 triple quadrupole mass spectrometer (AB Sciex, Foster City, CA, USA) coupled to a Shimadzu LC (SLC-10A VP) system (Shimadzu, Columbia, MA, USA) and an HTS PAL LEAP autosampler (LEAP Technologies, Morrisville, NC, USA). Details of the procedure, the used peptides and mass transitions have been previously described elsewhere [38].

### 2.3. Statistical Methods

The distribution of the analyzed variables was tested for normality using Shapiro–Wilk test. Differences between the study groups were evaluated using a non-parametric Kruskal–Wallis test for multiple comparisons followed by a post hoc Dunn’s test. Data marked as ‘ns’ are the results of the Kruskal–Wallis test with a *p*-value greater than 0.05. All calculations were performed using the Statistica 13.3 software package (TIBCO Software Inc., Palo Alto, CA, USA). Values of *p* < 0.05 were considered statistically significant.

## 3. Results

Significantly lower levels of both *UGTA1A1* gene expression and UGT1A1 protein content were observed in livers from subjects with the *28/28 genotype compared to patients with the *1/1 genotype. These differences were significant in the control group, as well as in patients with liver disease. After the patients were stratified by the Child–Pugh classification system, significant genotype-related differences measured in protein level were only noted in patients with class B.

**Figure 1:**
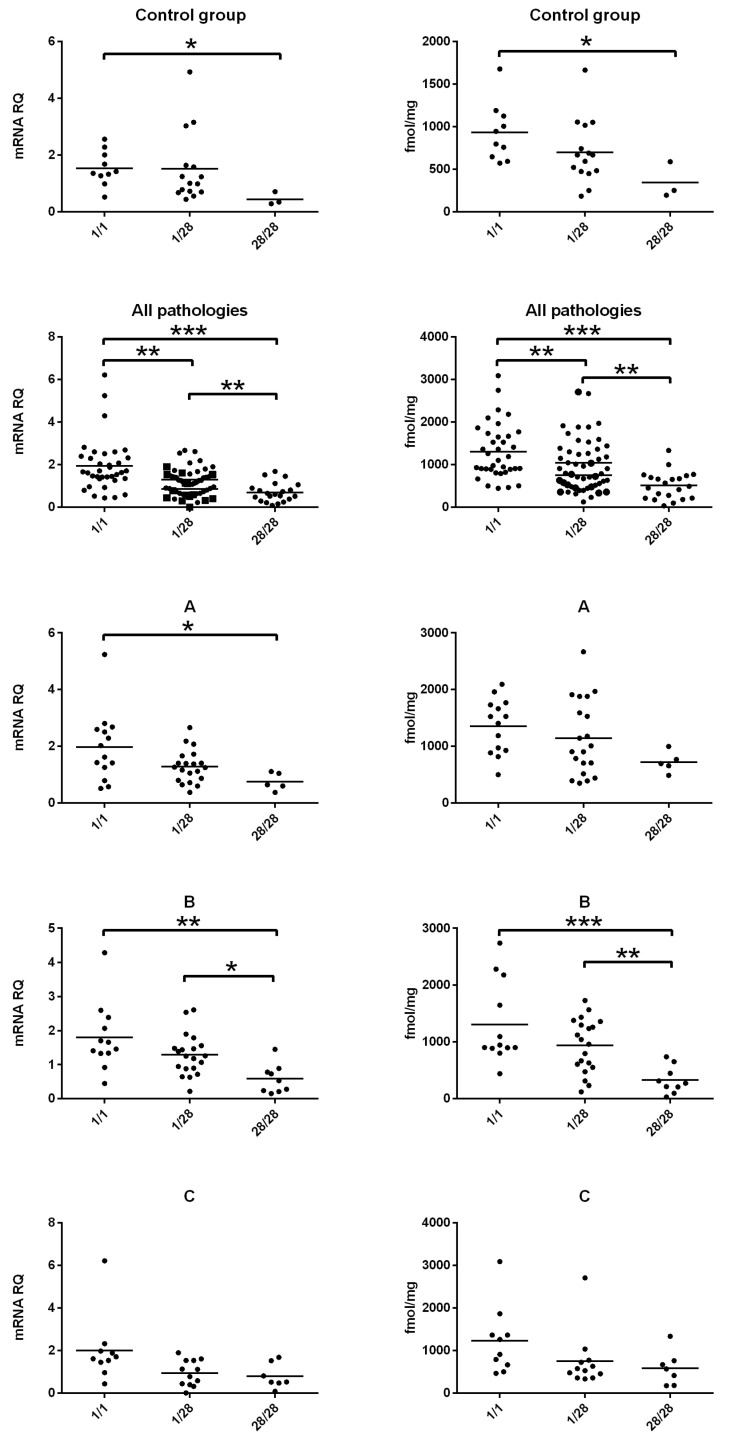
Gene expression (**left**) and protein abundance (**right**) of UGT1A1 in relation to *UGT1A1* genotype. Data are presented as mean ± standard deviation (SD). mRNA level of the analyzed genes was expressed as a relative amount to the mean expression of the housekeeping genes (*GAPDH*, *HMBS*, *PPIA*, *RPLP0*, *RPS9*). RQ—relative gene expression levels determined by the comparative ΔCt method. A, B, C—data presented for different stages of liver damage assessed with the Child–Pugh scale. Statistically significant differences: * *p* < 0.05, ** *p* < 0.01, *** *p* < 0.001 (Kruskal–Wallis test with post hoc Dunn’s test) between subjects with different *UGT1A1* genotypes.

Gene expression and protein content were intermediate in patients with the heterozygous *1/*28 genotype. The difference between heterozygotes and *1/*1 or *28/*28 homozygotes was significant only in some subgroups of the study, as illustrated in Figure 1 and Supplementary Table S3.

Similar significant genotype-dependent differences were observed when liver tissue samples from patients with distinct liver diseases were analyzed separately for AIH, HCV, ALD and PBC. However, a reliable analysis of gene expression (mRNA) or protein content of UGT1A1 in PSC and WD could not be performed due to the number of samples in the subgroups being too low (Table 1). Due to insufficient statistical power, the results obtained for distinct liver diseases should be considered exploratory only.

As UGT1A1 plays a major role in bilirubin conjugation, an additional analysis was performed to investigate the impact of the *UGT1A1* genotype on serum bilirubin concentration, given that data were available for most of the study subjects. Significant genotype-related differences in bilirubin concentration were only observed in the control group–patients without liver failure, where the mean concentrations were the lowest in patients with the *1/*1 genotype, intermediate in the *1/*28 subgroup, and the highest in *28/*28 homozygotes. No statistically significant differences in bilirubin concentration were observed in relation to genotype in patients with liver disease. Furthermore, no significant genotype-related differences were found in subgroups with different stages of liver failure (Child–Pugh classes A, B or C) or in any subgroup of patients with a specific liver disease (Table 2).

Additional information regarding the distribution of clinical characteristics in the study population is provided in Supplementary Table S4.

## 4. Discussion

The UGT1A1*28 polymorphism is the most common cause of Gilbert’s syndrome in Caucasians. It is characterized by an increased number of TA repeats in the gene promoter region, which results in reduced transcription of the gene and enzyme activity. Most studies investigating *UGT1A1* genetic variability have focused primarily on indirect measures of enzyme function, such as bilirubin concentration or glucuronidation assays. Direct assessment of UGT1A1 mRNA and protein expression in human liver tissue remains limited due to restricted access to biological material. Furthermore, previous studies have rarely evaluated these parameters in the context of chronic liver disease, despite the fact that hepatic pathology itself may influence the regulation of drug-metabolizing enzymes. Consequently, the extent to which liver disease modifies the relationship between *UGT1A1* genotype and gene expression at the transcript and protein levels remains unclear.

The results of the current study are consistent with the observations of Girard et al. [29], who analyzed the effect of the UGT1A1*28 polymorphism on the UGT1A1 protein level and enzyme activity in the human liver. The authors demonstrated that the presence of the *28 allele was associated with a significant reduction in UGT1A1 protein content, as well as a decrease in the enzyme’s ability to conjugate the carcinogenic metabolite (N-OH-PhIP). An increase in the genotype effect was observed depending on the increasing number of *28 alleles (*1/*1 > *1/*28 > *28/*28), whereas the significant decrease in both protein content and enzymatic activity was most pronounced in *28/*28 homozygotes. The authors stated that those results may suggest that the UGT1A1*28 polymorphism could increase the risk of developing cancers associated with dietary exposure to heterocyclic amines. A similar reduction in *UGT1A1* expression was observed in patients with Gilbert’s syndrome in the current study.

Genotype-dependent reduction in mRNA expression and UGT1A1 protein level were also reported by Peterkin et al. [39]. However, the authors noted that the UGT1A1*28 polymorphism explains only around 40% of the variability in UGT1A1 activity between individuals, suggesting the existence of other factors that modulate enzyme function. Enzyme activity can also be influenced by post-translational modifications, or environmental factors that are independent of gene expression. This may partly explain the inconsistency observed in the current study: relation between *UGT1A1* genotype and bilirubin concentration was clear and significant in the control group, while we did not report that in case of patients with liver disease, nor in the subgroups of patients stratified by the specific liver disease or Child–Pugh class. Moreover, no statistically significant sex-related differences in bilirubin levels were observed among patients with liver disease. In contrast, bilirubin concentrations in the control group were associated with sex, although they did not differ significantly between the analyzed genotypes. These results suggest that metabolic changes in advanced liver disease can overwhelm genetic predisposition.

In the past, bilirubin was mainly considered to be a potentially toxic degradation product with no specific physiological function. More recently it was presented that bilirubin has antioxidant, anti-inflammatory, and immunomodulatory properties. As a consequence, slightly elevated bilirubin concentration observed in Gilbert’s syndrome has a protective effect against conditions like diabetes, cardiovascular disorders, metabolic syndrome, or liver disease [40]. However, further investigation is needed to verify if Gilbert’s syndrome is related to modified progression of liver disease, as the design of the current study does not allow for such an analysis.

Similarly, the genotype impact related to the number of UGT1A1*28 alleles was also confirmed by Yoder Graber et al. study [30], who investigated thyroxine glucuronidation rate by the UGT1A1 enzyme (*1/*1 > *1/*28 > *28/*28). Their results confirmed that the presence of the *28 polymorphism leads to reduced *UGT1A1* expression. The authors indicated the possible clinical consequences of alteration in enzyme activity–patients with the *28/28 genotype may achieve higher thyroxine concentrations with standard doses of medication in the course of hypothyroidism treatment. These observations highlight the broader significance of the UGT1A1*28 polymorphism in relation to not only bilirubin and cytostatic drug metabolism, but also commonly used substitution therapy. This gives the results of the study a broader clinical application.

Liu et al. [41] assessed the impact of various genetic variants in the promoter region on the expression and activity of UGT1A1. Unlike the present study, which was focused exclusively on detecting allele *28, the authors also considered approximately 43 other variants within the *UGT1A* locus. Despite the broader analysis, it was the *28 allele that showed the strongest association with reduced mRNA expression and decreased enzyme activity. Homozygotes with the genotype determining Gilbert’s syndrome (*28/*28) were found to have significantly lower levels of gene expression. The authors also examined the relationship between UGT1A1 expression and several transcription factors and identified CAR (constitutive androstane receptor), PXR (pregnane X receptor), and ESR1 (estrogen receptor 1) as the most significant regulators of UGT transcription. The affinity of these transcription factors for promoter sequences may contribute to the differences in UGT1A1 gene expression and protein content related to the presence of the *28 allele, which was also observed in the present study. While the *28 allele appears to be the main determinant of reduced gene expression and protein content, literature data suggest that other promoter variants may also significantly reduce *UGT1A1* gene expression efficiency. Nevertheless, the *28 allele remains the most common functional variant allele in Caucasians, while other alleles are rare or in close linkage disequilibrium with UGT1A1*28 [42,43].

The current study demonstrated that progressive liver failure, as assessed by the Child–Pugh scale, had no significant impact on the expression of the *UGT1A1* gene in groups with the same genotype. This suggests that genetic variants, rather than the severity of liver failure itself, are the dominant cause of altered enzyme expression. This finding is consistent with previous studies indicating that the Child–Pugh classification system does not affect the expression of certain drug-metabolizing enzymes, including UGT1A1. Unlike some CYP450 enzymes, the content of UGT1A1 protein does not decrease with the progression of liver failure as assessed based on the Child–Pugh classification [34,44]. However, it is worth emphasizing that the Child–Pugh score is a relatively crude clinical index and may not capture molecular-level changes in hepatocyte function [45].

Furthermore, the current research observed a reduction in *UGT1A1* expression in relation to the UGT1A1*28 polymorphism in livers from patients with various liver diseases of different etiology. In autoimmune hepatitis (AIH) and hepatitis C (HCV) livers, *UGT1A1* expression was significantly lower at both the mRNA and protein level (*p* < 0.03). In the context of AIH, Bachrich et al. [46] reported the presence of autoantibodies against UGT1A1 in patients with type 2 AIH in an in vitro study. While disease subtypes and autoantibody status were not evaluated in the present analysis, the existence of genotype-dependent effects suggests that potential autoimmune responses do not substantially alter UGT1A1 transcription or translation. However, as enzyme activity was not examined, the in vivo impact of autoantibodies on UGT1A1 function cannot be excluded. In patients with HCV, no significant association has been reported between the *UGT1A1* genotype and the stage of liver fibrosis or the presence of cirrhosis [47,48]. Consistently, the present study found no significant difference in the frequency of *UGT1A1* genotypes between patients and controls, or among subgroups stratified by the severity of liver failure.

In alcohol liver disease (ALD) and primary biliary cholangitis (PBC), the relationship between the *UGT1A1* genotype and its expression level was less clear than in other groups. However, in both cases, significant differences were demonstrated between selected genotypes. Recent literature suggests that *UGT1A1* genetic variants may modify the clinical manifestations of PSC by affecting bilirubin and bile acid metabolism under cholestatic conditions. A reported case of PSC with a concomitant *UGT1A1* mutation and recurrent bile duct stones indicates that impaired glucuronidation may contribute to atypical biliary complications [49]. Although UGT1A1 is not considered a factor predisposing to PSC, altered enzyme function may influence the disease’s specific clinical features. However, due to a limited number of patients in subgroups, the preliminary results obtained for distinct liver diseases in the current study should be treated with caution.

Additionally, presented results should also be interpreted in the context of disease-related regulatory mechanisms that may affect hepatic *UGT1A1* expression independently of genetic background. Chronic liver diseases may substantially influence *UGT1A1* expression through inflammatory processes, fibrosis, or hepatocellular remodeling, altering transcriptional regulation and protein synthesis [50]. In particular, pro-inflammatory cytokines may modulate *UGT1A1* expression via altered activity of nuclear receptors such as constitutive androstane receptor (CAR) and pregnane X receptor (PXR), which are key regulators of UGT transcription [51]. Furthermore, the analyzed liver diseases differ considerably in their underlying mechanisms, which may contribute to the expression patterns independently of genetic variation.

Although the results of the current study are consistent with previous findings, the study has several limitations. UGT1A1 activity is influenced not only by genetic variants but also by other factors, meaning that the expression does not fully reflect the enzyme’s functional metabolic capacity [52]. In the present study, *UGT1A1* expression was only assessed at the mRNA and protein levels. Direct measurement of enzymatic activity was not possible, which limits the ability to fully evaluate the impact of the *28 allele on the metabolism rate of UGT1A1 substrates. Therefore, it is difficult to draw clear conclusions about the impact of genotype on the metabolism of drugs that are enzyme substrates (such as irinotecan, levothyroxine, acetaminophen or belinostat). Additionally, in the case of some diseases (PSC, WD), the number of samples was too small to perform a reliable statistical analysis, and this needs to be expanded in future research. In summary, the *UGT1A1* expression patterns observed in the analyzed groups are a component of many endogenous and exogenous factors, which, together with the genetic profile, contribute to the overall picture of reduced UGT1A1 expression in control tissues and tissues from patients with various liver diseases.

In the current study, a non-tumorous tissue from metastatic colon cancer patients was used as a control. Since it was demonstrated that low bilirubin levels can be associated with elevated risk of several cancer types, both bilirubin levels and Gilbert’s syndrome prevalence in that group may not reflect the values in the general healthy population [53]. However, we have previously conducted a preliminary study, comparing donor livers and non-tumorous tissue from metastatic livers, and no significant differences in *UGT1A1* expression (measured both at mRNA and protein content) were observed [54].

Despite the indicated limitations, the obtained results confirm the impact of the UGT1A1*28 polymorphism on hepatic gene expression in patients with liver disease.

## 5. Conclusions

Patients with Gilbert’s syndrome (UGT1A1*28/*28) are characterized by lower *UGT1A1* gene expression at both the mRNA and protein level, regardless of the pathology type or liver failure stage. The effect of UGT1A1*28 may potentially contribute to the metabolism of its substrates, including drugs. However, its relative importance compared to concomitant liver disease and its consequences, i.e., metabolic and structural alterations, remains uncertain.

## Figures and Tables

**Table 1:** Gene expression quantity and protein content of the UGT1A1 in relation to *UGT1A1* genotype in controls and patients with different liver diseases.

Group	Parameter	Genotype			*p*-Value		
		*1/*1	*1/*28	*28/*28	*1/*28 vs. *28/*28	*1/*28 vs. *1/*1	*1/*1 vs. *28/*28
Control	*n*	10	15	3			
	RQ	1.54 ± 0.58	1.51 ± 1.21	0.44 ± 0.19	0.121	0.825	0.024
	protein	930.18 ± 322.75	699.92 ± 361.21	344.40 ± 174.03	0.373	0.175	0.035
AIH	*n*	7	4	6	*p*		
	RQ	2.11 ± 1.61	0.73 ± 0.81	0.55 ± 0.56	1.000	0.327	0.049
	protein	1652.43 ± 677.64	664.52 ± 260.39	475.32 ± 228.93	1.000	0.123	0.004
ALD	*n*	6	8	5	*p*		
	RQ	1.77 ± 0.46	1.167 ± 0.70	0.62 ± 0.59	0.540	0.267	0.016
	protein	877.33 ± 257.78	383.38 ± 177.22	522.76 ± 514.03	1.000	0.034	0.23
HCV	*n*	16	33	8	*p*		
	RQ	2.20 ± 1.42	1.36 ± 0.54	0.86 ± 0.32	0.093	0.082	0.001
	protein	1286.18 ± 606.32	1201.07 ± 608.50	546.75 ± 304.13	0.012	1.000	0.008
PBC	*n*	5	4	2	*p*		
	RQ	1.40 ± 0.10	0.56 ± 0.26	0.68 ± 0.30	1.000	0.021	0.315
	protein	1426.06 ± 950.36	488.47 ± 89.44	414.78 ± 336.32	1.000	0.040	0.142
PSC	*n*	2	3	0	*p*		
	RQ	0.93 ± 0.58	1.16 ± 0.10	nd	nd	1.000	nd
	protein	1212.33 ± 444.36	1095.77 ± 367.54	nd	nd	0.773	nd
WD	*n*	3	3	0	*p*		
	RQ	0.96 ± 0.39	0.48 ± 0.14	nd	nd	0.081	nd
	protein	852.67 ± 257.74	1014.36 ± 502.59	nd	nd	1.000	nd

Gene expression and protein abundance in different liver pathologies: hepatic tissues from patients with hepatitis C (HCV), primary biliary cholangitis (PBC), primary sclerosing cholangitis (PSC), alcoholic liver disease (ALD), Wilson’s disease (WD) and autoimmune hepatitis (AIH). All results of protein content are given in fmol/mg of the analyzed tissue. RQ was determined using the comparative ΔCt method.

**Table 2:** Bilirubin concentration in study subjects in control group and in liver disease.

Group	*n*	Bilirubin Serum Concentration [mg/dL]				*p*-Value		
		All Groups	*1/*1	*1/*28	*28/*28	*1/*28 vs. *28/*28	*1/*28 vs. *1/*1	*1/*1 vs. *28/*28
All pathologies	112	3.847 ± 9.017	3.074 ± 3.315	4.542 ± 12.749	3.491 ± 3.554	ns	ns	ns
Control group	22	0.636 ±0.51	0.271 ± 0.066	0.639 ± 0.298	1.473 ± 0.665	ns	0.021	0.004
HCV	56	1.678 ± 1.214	1.787 ± 1.498	1.804 ± 1.227	1.444 ± 0.816	ns	ns	ns
ALD	19	4.615 ± 4.275	4.692 ± 4.578	2.301 ± 1.099	8.224 ± 4.137	ns	ns	ns
AIH	18	3.165 ± 3.665	1.282 ± 0.707	6.855 ± 5.323	2.895 ± 1.778	ns	ns	ns
PBC	10	5.895 ± 6.04	1.950 ± 1.461	10.767 ± 8.262	4.385 ± 1.685	ns	ns	ns
PSC	5	5.366 ± 6.477	5.000 ± 2.700	5.610 ± 7.136	nd	ns	ns	ns
WD	5	21.91 ± 38.527	8.135 ± 7.035	31.093 ± 41.657	nd	ns	ns	ns
Child – Pugh A	39	1.046 ± 0.533	1.028 ± 0.628	1.050 ± 0.569	0.890 ± 0.116	ns	ns	ns
Child – Pugh B	39	2.717 ± 2.634	2.465 ± 0.637	2.809 ± 3.256	2.730 ± 1.491	ns	ns	ns
Child – Pugh C	29	6.018 ± 4.965	4.586 ± 3.576	6.572 ±5.887	7.113 ± 4.033	ns	ns	ns

Bilirubin levels are presented as mean ± SD. ns—not significant (Kruskal–Wallis test); nd—no data.

## Data Availability

The original contributions presented in this study are included in the article/Supplementary Materials. Further inquiries can be directed to the corresponding author.

## Supplementary Materials

The following supporting information can be downloaded at: https://www.mdpi.com/article/10.3390/genes17050589/s1. Table S1: Characteristics of the subjects; Table S2: qPCR assays; Table S3: RQ and protein content in the Child–Pugh scale subgroups; Table S4: Distribution of major clinical characteristics.

## Abbreviations

The following abbreviations are used in this manuscript:

UGT	UDP-glucuronosyltransferase
HCV	Hepatitis C virus
ALD	Alcoholic liver disease
PSC	Primary sclerosing cholangitis
PBC	Primary biliary cholangitis
AIH	Autoimmune hepatitis
WD	Wilson’s disease
