# Supplementary material for: The Impact of UGT1A1 Genetic Variability on Enzyme Expression in Liver Pathology
Source: Genes (Basel). 2026 May 21;17(5):589. doi: 10.3390/genes17050589 (PMC13205437; doi:10.3390/genes17050589)
Supplement: Supplementary file 1 [file genes-17-00589-s001.zip › genes-4265426-supplementary.pdf]

Table S1. Characteristics of the subjects (mean ± SD).

	Control Goup	HCV	PBC	PSC	ALD	AIH	WD
**N**	28	57	11	5	19	17	6
**Female (%)**	39.3 %	49.1%	90.9%*	20.0%	21.1%	58.8%	33.3%
**Age (mean ± SD)**	58.0±14.0	56.0±7.4	59.0±4.5	41.2±11.7	51.2±6.5	46.2±6.6	34.3±9.9
**Child-Pugh class (A/B/C)**	ND	29/21/7	2/4/5	3/2/0	0/8/11	5/6/6	1/2/3

Table S2. List of assays used for mRNA quantification.

Gene Symbol	Assay ID (Thermo Fisher Scientific)
UGT1A1	Hs02511055_s1
GAPDH	Hs99999905_m1
HMBS	Hs00609297_m1
PPIA	Hs99999904_m1
RPLP0	Hs99999902_m1
RPS9	Hs02339424_g1

Table S3. Relative gene expression and protein content of the UGT1A1 in relation to *UGT1A1* genotype in control group. subjects with liver disease, subsequently subdivided based on degree of liver failure assessed by means of the Child-Pugh score.

**Group**		**Genotype**			***p*-Value**		
		***1/*1**	***1/*28**	***28/*28**	***1/*28 vs *28/*28**	***1/*28 vs *1/*1**	***1/*1 vs *28/*28**
**Control**	**n**	10	15	3			
	**RQ**	1.54 ± 0.58	1.51 ± 1.21	0.44 ± 0.19	0.121	0.825	0.024
	**protein**	930.18 ± 322.75	699.92 ± 361.21	344.40 ± 174.03	0.372	0.275	0.035
**All pathologies**	**n**	36	52	21			
	**RQ**	1.93 ± 1.20	1.21 ± 0.59	0.70 ± 0.44	0.002	0.005	<0.001
	**protein**	1304.58 ± 633.56	973.11 ± 5925.32	508.06 ± 325.30	0.001	0.014	<0.001
**A**	**n**	14	20	5			
	**RQ**	1.98 ± 1.17	1.29 ± 0.56	0.76 ± 0.28	0.217	0.252	0.012
	**protein**	1355.66 ± 460.97	1143.28 ± 644.90	722.07 ± 165.95	ns	ns	ns
**B**	**n**	12	20	9			
	**RQ**	1.80 ± 0.94	1.29 ± 0.59	0.59 ± 0.40	0.024	0.478	0.001
	**protein**	1311.65 ± 691.85	940.27 ± 460.07	330.64 ± 226.38	0.006	0.879	<0.001
**C**	**n**	10	12	7			
	**RQ**	2.00 ± 1.49	0.94 ± 0.59	0.80 ± 0.54	1.000	0.074	0.068
	**protein**	1224.57 ± 750.37	744.24 ± 622.38	583.30 ± 371.39	1.000	0.102	0.089

All results of protein content are given in fmol/mg of the analyzed tissue. A, B, C - degree of liver failure measured with Child-Pugh scale. ns – not significant in Kruskal-Wallis test

Table S4. Distribution of major clinical characteristics across comparison groups.

Group	Albumin Serum Concentration [mg/dL]				Age				Sex (Male/Female)		
	n	*1/*1	*1/*28	*28/*28	n	*1/*1	*1/*28	*28/*28	*1/*1	*1/*28	*28/*28
**Control group**	19	3.30 ± 0.94	3.70 ± 0.58	4.95 ± 1.45	28	57 ± 12	60 ± 11	52 ± 23	5/5	9/6	3/0
**HCV**	57	3.27± 0.58	3.39± 0.53	3.54± 0.64	57	56 ± 9	56 ± 7	56 ± 5	7/9	19/14	3/5
**ALD**	19	2.73± 0.36	3.13± 0.53	3.12± 0.52	19	49 ± 6	51 ± 6	54 ± 5	5/1	7/1	3/2
**AIH**	19	3.57± 0.21	3.03± 0.35	3.40± 0.35	17	44 ± 14	31 ± 5	59 ± 13	2/5	2/2	3/3
**PBC**	10	2.93± 0.69	3.41± 0.58	2.90± 0.20	11	61 ± 4	55 ± 1	62 ± 3	1/4	0/4	0/2
**PSC**	5	3.40± 0.20	3.90± 0.44	nd	5	47 ± 12	38 ± 8	nd	2/0	2/1	nd
**WD**	4	3.50± 0.40	3.30± 0.20	nd	6	39 ± 5	30 ± 10	nd	2/1	2/1	nd
**Child-Pugh A**	39	3.51± 0.49	3.80± 0.39	3.94± 0.38	39	56 ± 7	54 ± 10	53 ± 11	6/8	9/11	3/2
**Child-Pugh B**	40	3.29± 0.49	3.21± 0.47	3.29± 0.52	41	47 ± 15	54 ± 8	57 ± 7	7/5	15/5	2/7
**Child-Pugh C**	29	2.74± 0.36	2.91± 0.36	2.97± 0.26	29	55 ± 8	47 ± 11	60 ± 6	4/6	6/6	4/3

Data are presented as mean ± SD.
